# [5-Hydroxy-3-phenyl-1-(pyridin-2-yl)pyrazol-5-olato]diphenylboron

**DOI:** 10.1107/S1600536811010634

**Published:** 2011-03-26

**Authors:** Kee-In Lee, Hye-Rin Bin, Do-Min Lee, Chong-Hyeak Kim

**Affiliations:** a Green Chemistry Division, Korea Research Institute of Chemical Technology, PO Box 107, Yuseong, Daejeon 305-600, Republic of Korea; b Center for Chemical Analysis, Korea Research Institute of Chemical Technology, PO Box 107, Yuseong, Daejeon 305-600, Republic of Korea

## Abstract

In the title compound, C_26_H_20_BN_3_O, the B atom has tetra­hedral geometry and is linked to two phenyl rings, the O atom of the hy­droxy­pyrazole ring and the N atom of the pyridinyl ring. A six-membered BOCNCN ring forms by coordination of the B atom and the pyridinyl N atom. The BOCNCN ring has an envelope conformation [dihedral angle = 36.7 (1)° between the planar ring atoms and the flap] with the B atom out of the plane. In the 1-(2-pyridin­yl)-3-phenyl-5-hy­droxy­pyrazole group, the pyridinyl ring, the phenyl ring and the pyrazole ring are almost coplanar: the pyrazole ring makes a dihedral angle of 9.56 (8)° with the pyridinyl ring and 17.68 (7)° with the phenyl ring. The crystal structure is stabilized by π–π stacking inter­actions involving the pyridinyl and pyrazole rings of centrosymmetrically related mol­ecules, with ring centroid separations of 3.54 (5) Å.

## Related literature

For general synthesis of diaryl­borinates, see: Hagan *et al.* (2000 ▶). For their synthesis and biological applications, see: Scorei & Popa (2010 ▶); Baker, Akama *et al.* (2006 ▶); Baker, Zhang *et al.* (2006 ▶). For luminescent organoboron compounds, see: Cui *et al.* (2005 ▶).
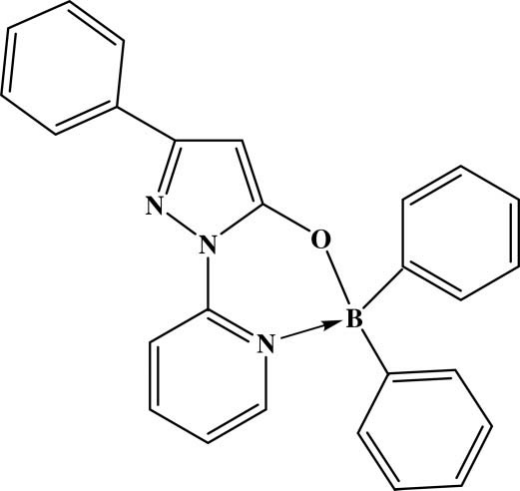


## Experimental

### 

#### Crystal data

C_26_H_20_BN_3_O
*M* *_r_* = 401.26
Triclinic, 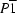

*a* = 9.7309 (1) Å
*b* = 9.8830 (1) Å
*c* = 11.2162 (1) Å
α = 78.966 (1)°
β = 81.795 (1)°
γ = 79.993 (1)°
*V* = 1035.91 (2) Å^3^
*Z* = 2
Mo *K*α radiation
μ = 0.08 mm^−1^
*T* = 296 K
0.44 × 0.31 × 0.23 mm

#### Data collection

Bruker APEXII CCD diffractometer
19866 measured reflections
5044 independent reflections
3923 reflections with *I* > 2σ(*I*)
*R*_int_ = 0.019

#### Refinement

*R*[*F* ^2^ > 2σ(*F* ^2^)] = 0.041
*wR*(*F* ^2^) = 0.106
*S* = 1.03
5044 reflections
280 parameters
H-atom parameters constrained
Δρ_max_ = 0.21 e Å^−3^
Δρ_min_ = −0.19 e Å^−3^

### 

Data collection: *APEX2* (Bruker, 2009 ▶); cell refinement: *SAINT* (Bruker, 2009 ▶); data reduction: *SAINT*; program(s) used to solve structure: *SHELXS97* (Sheldrick, 2008 ▶); program(s) used to refine structure: *SHELXL97* (Sheldrick, 2008 ▶); molecular graphics: *XP* in *SHELXTL* (Sheldrick, 2008 ▶); software used to prepare material for publication: *SHELXL97*.

## Supplementary Material

Crystal structure: contains datablocks global, I. DOI: 10.1107/S1600536811010634/pk2309sup1.cif

Structure factors: contains datablocks I. DOI: 10.1107/S1600536811010634/pk2309Isup2.hkl

Additional supplementary materials:  crystallographic information; 3D view; checkCIF report

## Figures and Tables

**Fig. 1.**
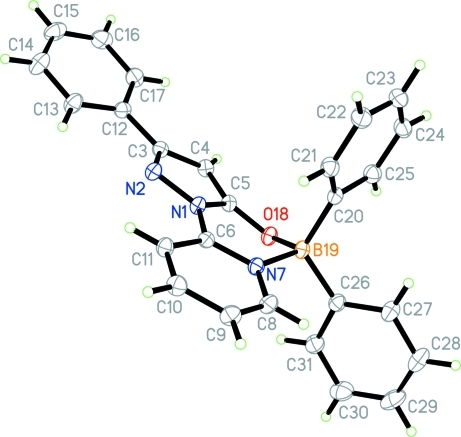
The molecular structure of the title compound with 30% probability displacement ellipsoids. H atoms are shown as small spheres of arbitary radius.

**Fig. 2.**
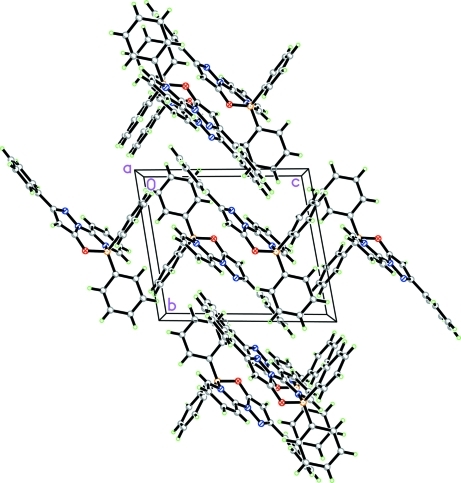
The molecular packing structure of the title compound along the a axis. [Symmetry codes: (i) x, y, z; (ii) -x, -y, -z.]

## supplementary crystallographic information

### Comment

Recently, boron-containing compounds have become increasingly frequent in preventive and chemotherapeutic agents for high-malignancy cancer (Scorei & Popa, 2010), their antibacterial and antifungal agents effect on cell growth (Baker, Akama *et al.*, 2006; Baker, Zhang *et al.*, 2006), and also in luminescent compounds for potential applications in organic light emitting devices (Cui *et al.*, 2005). Diarylborinates have been prepared by the reaction of diarylborinic acids with dimethylaminoethanol-type ligands (Hagan *et al.*, 2000). Interestingly, we identify an unusual diphenylborinate from 1-(2-pyridinyl)pyrazole trifluoromethanesulfonate with phenylboronic acid in Suzuki-Miyaura coupling conditions. Here we report the crystal structure of the title compound (Figs. 1 and 2). In the title compound, C_26_H_20_BN_3_O, B atom has a tetrahedral geometry linked with the two C atoms of the two phenyl rings, the O atom of the hydroxypyrazole ring and the N atom of the pyridinyl ring. The six membered ring of –B(19)—O(18)—C(5)—N(1)—C(6)—N(7)- motif forms by the coordination between B atom and N atom of the pyridinyl ring. The –B—O—C—N—C—N– ring has an envelope conformation with B out of plane. The B atom deviate from the the mean plane and forms a dihedral angle of 36.7 (1) ° with B(19)—O(18) 1.508 (2) Å and B(19)—N(7) 1.641 (2) Å. In the 1-(2-pyridinyl)-3-phenyl-5-hydroxypyrazole group the pyridinyl ring, the phenyl ring and the pyrazole ring are almost coplanar. The pyrazole ring makes a dihedral angle of 9.56 (8)° with the pyridinyl ring, and 17.68 (7)° with the phenyl ring. The crystal structure is stabilized by π-π stacking interactions involving the pyridinyl and pyrazole rings of centrosymmetrically related molecules, with a ring centroid separations of 3.54 (5) Å.

### Experimental

The title compound was synthesized from the reaction of 1-(2-pyridinyl)pyrazole trifluoromethanesulfonate (185 mg, 0.5 mmol) and phenylboronic acid (183 mg, 1.5 mmol) with K_3_PO_4_ (318 mg, 1.5 mmol) in the presence of PdCl_2_{1,1'-bis(diphenylphosphino)ferrocene} (33 mg, 0.04 mmol) and 1,1'-bis(diphenylphosphino)ferrocene (11 mg, 0.02 mmol) in anhydrous 1,4-dioxane (5 ml) for 20 hr at 100 °C. The reaction mixture was rinsed with toluene (5 ml) and the resulting solution was filtered by aid of a Celite pad. The organic layer was dried over Na_2_SO_4_ and the solvents were removed. The residue was purified by column chromatography on silica gel (hexane/ethyl acetate = 7/1) to give the title compound (30 mg, 8% yield). Single crystals suitable for X-ray diffraction were prepared by slow evaporation of a solution in ethyl acetate at room temperature.

### Refinement

All H atoms were placed in calculated positions using a riding model, with C—H = 0.93 Å and *U*_iso_(H) = 1.2 *U*_eq_(C).

### Crystal data

**Table:**

C_26_H_20_BN_3_O	*Z* = 2
*M**_r_* = 401.26	*F*(000) = 420
Triclinic, *P*1	*D*_x_ = 1.286 Mg m^−^^3^
Hall symbol: -P 1	Mo *K*α radiation, λ = 0.71073 Å
*a* = 9.7309 (1) Å	Cell parameters from 8036 reflections
*b* = 9.8830 (1) Å	θ = 2.6–27.8°
*c* = 11.2162 (1) Å	µ = 0.08 mm^−^^1^
α = 78.966 (1)°	*T* = 296 K
β = 81.795 (1)°	Block, pale-yellow
γ = 79.993 (1)°	0.44 × 0.31 × 0.23 mm
*V* = 1035.91 (2) Å^3^	

### Data collection

**Table:**

Bruker APEXII CCD diffractometer	3923 reflections with *I* > 2σ(*I*)
Radiation source: fine-focus sealed tube	*R*_int_ = 0.019
graphite	θ_max_ = 28.2°, θ_min_ = 1.9°
φ and ω scans	*h* = −12→12
19866 measured reflections	*k* = −13→13
5044 independent reflections	*l* = −14→14

### Refinement

**Table:**

Refinement on *F*^2^	Primary atom site location: structure-invariant direct methods
Least-squares matrix: full	Secondary atom site location: difference Fourier map
*R*[*F*^2^ > 2σ(*F*^2^)] = 0.041	Hydrogen site location: inferred from neighbouring sites
*wR*(*F*^2^) = 0.106	H-atom parameters constrained
*S* = 1.02	*w* = 1/[σ^2^(*F*_o_^2^) + (0.0466*P*)^2^ + 0.1687*P*] where *P* = (*F*_o_^2^ + 2*F*_c_^2^)/3
5044 reflections	(Δ/σ)_max_ < 0.001
280 parameters	Δρ_max_ = 0.21 e Å^−^^3^
0 restraints	Δρ_min_ = −0.19 e Å^−^^3^

### Special details

**Table:**

Geometry. All e.s.d.'s (except the e.s.d. in the dihedral angle between two l.s. planes) are estimated using the full covariance matrix. The cell e.s.d.'s are taken into account individually in the estimation of e.s.d.'s in distances, angles and torsion angles; correlations between e.s.d.'s in cell parameters are only used when they are defined by crystal symmetry. An approximate (isotropic) treatment of cell e.s.d.'s is used for estimating e.s.d.'s involving l.s. planes.
Refinement. Refinement of *F*^2^ against ALL reflections. The weighted *R*-factor *wR* and goodness of fit *S* are based on *F*^2^, conventional *R*-factors *R* are based on *F*, with *F* set to zero for negative *F*^2^. The threshold expression of *F*^2^ > σ(*F*^2^) is used only for calculating *R*-factors(gt) *etc*. and is not relevant to the choice of reflections for refinement. *R*-factors based on *F*^2^ are statistically about twice as large as those based on *F*, and *R*- factors based on ALL data will be even larger.

### Fractional atomic coordinates and isotropic or equivalent isotropic displacement parameters (Å^2^)

**Table:**

	*x*	*y*	*z*	*U*_iso_*/*U*_eq_	
N1	0.34223 (10)	0.62671 (9)	0.41391 (8)	0.0395 (2)	
N2	0.33209 (10)	0.73403 (10)	0.47857 (9)	0.0425 (2)	
C3	0.20130 (12)	0.74313 (12)	0.53352 (10)	0.0409 (2)	
C4	0.12724 (12)	0.64242 (12)	0.50700 (10)	0.0430 (3)	
H4A	0.0351	0.6293	0.5354	0.052*	
C5	0.22001 (11)	0.56935 (11)	0.43117 (9)	0.0386 (2)	
C6	0.46530 (11)	0.58301 (11)	0.34599 (9)	0.0367 (2)	
N7	0.45672 (9)	0.49060 (9)	0.27425 (8)	0.0384 (2)	
C8	0.57501 (13)	0.44224 (13)	0.20644 (11)	0.0468 (3)	
H8A	0.5706	0.3766	0.1583	0.056*	
C9	0.70060 (13)	0.48605 (13)	0.20602 (12)	0.0510 (3)	
H9A	0.7800	0.4516	0.1578	0.061*	
C10	0.70778 (13)	0.58275 (13)	0.27872 (12)	0.0495 (3)	
H10A	0.7922	0.6145	0.2790	0.059*	
C11	0.59005 (12)	0.63144 (12)	0.35022 (11)	0.0443 (3)	
H11A	0.5936	0.6953	0.4003	0.053*	
C12	0.15138 (13)	0.85394 (12)	0.60693 (10)	0.0443 (3)	
C13	0.24704 (15)	0.92376 (14)	0.64202 (13)	0.0583 (3)	
H13A	0.3428	0.8949	0.6246	0.070*	
C14	0.20174 (19)	1.03558 (16)	0.70253 (14)	0.0709 (4)	
H14A	0.2668	1.0816	0.7255	0.085*	
C15	0.05972 (19)	1.07890 (16)	0.72888 (14)	0.0706 (4)	
H15A	0.0288	1.1555	0.7678	0.085*	
C16	−0.03519 (17)	1.00873 (17)	0.69750 (13)	0.0681 (4)	
H16A	−0.1308	1.0368	0.7168	0.082*	
C17	0.00942 (14)	0.89644 (15)	0.63743 (12)	0.0568 (3)	
H17A	−0.0563	0.8490	0.6173	0.068*	
O18	0.21775 (8)	0.45786 (8)	0.38208 (7)	0.0434 (2)	
B19	0.30310 (14)	0.45262 (13)	0.25937 (12)	0.0403 (3)	
C20	0.23337 (12)	0.57188 (12)	0.15691 (10)	0.0415 (2)	
C21	0.29568 (15)	0.68316 (13)	0.08918 (11)	0.0519 (3)	
H21A	0.3863	0.6910	0.1015	0.062*	
C22	0.22721 (18)	0.78278 (15)	0.00390 (13)	0.0644 (4)	
H22A	0.2717	0.8562	−0.0395	0.077*	
C23	0.09335 (17)	0.77314 (15)	−0.01651 (13)	0.0641 (4)	
H23A	0.0470	0.8398	−0.0736	0.077*	
C24	0.02870 (15)	0.66393 (16)	0.04825 (13)	0.0595 (3)	
H24A	−0.0614	0.6563	0.0345	0.071*	
C25	0.09721 (13)	0.56573 (14)	0.13358 (12)	0.0512 (3)	
H25A	0.0515	0.4931	0.1770	0.061*	
C26	0.32831 (12)	0.29614 (12)	0.23273 (11)	0.0443 (3)	
C27	0.33295 (15)	0.26571 (15)	0.11599 (13)	0.0581 (3)	
H27A	0.3125	0.3381	0.0517	0.070*	
C28	0.36740 (19)	0.12997 (17)	0.09288 (17)	0.0760 (5)	
H28A	0.3698	0.1124	0.0140	0.091*	
C29	0.39796 (18)	0.02182 (16)	0.18673 (19)	0.0793 (5)	
H29A	0.4220	−0.0690	0.1713	0.095*	
C30	0.39304 (18)	0.04790 (15)	0.30304 (17)	0.0729 (4)	
H30A	0.4132	−0.0253	0.3668	0.087*	
C31	0.35801 (15)	0.18321 (13)	0.32561 (14)	0.0574 (3)	
H31A	0.3542	0.1993	0.4051	0.069*	

### Atomic displacement parameters (Å^2^)

**Table:**

	*U*^11^	*U*^22^	*U*^33^	*U*^12^	*U*^13^	*U*^23^
N1	0.0393 (5)	0.0417 (5)	0.0400 (5)	−0.0107 (4)	0.0002 (4)	−0.0123 (4)
N2	0.0449 (5)	0.0423 (5)	0.0429 (5)	−0.0092 (4)	−0.0006 (4)	−0.0145 (4)
C3	0.0414 (6)	0.0433 (6)	0.0367 (5)	−0.0053 (5)	−0.0029 (4)	−0.0059 (5)
C4	0.0390 (6)	0.0503 (6)	0.0400 (6)	−0.0101 (5)	−0.0007 (4)	−0.0082 (5)
C5	0.0409 (6)	0.0411 (6)	0.0348 (5)	−0.0114 (4)	−0.0039 (4)	−0.0046 (4)
C6	0.0394 (6)	0.0363 (5)	0.0334 (5)	−0.0067 (4)	−0.0031 (4)	−0.0037 (4)
N7	0.0410 (5)	0.0390 (5)	0.0358 (5)	−0.0082 (4)	−0.0018 (4)	−0.0078 (4)
C8	0.0480 (7)	0.0477 (6)	0.0452 (6)	−0.0070 (5)	0.0017 (5)	−0.0148 (5)
C9	0.0429 (6)	0.0553 (7)	0.0527 (7)	−0.0059 (5)	0.0055 (5)	−0.0132 (6)
C10	0.0397 (6)	0.0542 (7)	0.0548 (7)	−0.0129 (5)	−0.0015 (5)	−0.0075 (6)
C11	0.0433 (6)	0.0468 (6)	0.0452 (6)	−0.0119 (5)	−0.0033 (5)	−0.0102 (5)
C12	0.0490 (6)	0.0443 (6)	0.0377 (6)	−0.0037 (5)	−0.0008 (5)	−0.0083 (5)
C13	0.0558 (8)	0.0589 (8)	0.0641 (8)	−0.0105 (6)	0.0038 (6)	−0.0259 (7)
C14	0.0851 (11)	0.0630 (9)	0.0709 (10)	−0.0198 (8)	0.0069 (8)	−0.0306 (8)
C15	0.0924 (12)	0.0559 (8)	0.0577 (8)	0.0035 (8)	0.0082 (8)	−0.0211 (7)
C16	0.0624 (9)	0.0760 (10)	0.0573 (8)	0.0126 (8)	0.0042 (7)	−0.0194 (7)
C17	0.0503 (7)	0.0692 (9)	0.0492 (7)	−0.0017 (6)	−0.0006 (6)	−0.0165 (6)
O18	0.0475 (5)	0.0454 (4)	0.0407 (4)	−0.0170 (3)	0.0010 (3)	−0.0111 (3)
B19	0.0418 (7)	0.0426 (7)	0.0391 (6)	−0.0122 (5)	−0.0031 (5)	−0.0094 (5)
C20	0.0468 (6)	0.0417 (6)	0.0384 (6)	−0.0071 (5)	−0.0029 (5)	−0.0137 (5)
C21	0.0604 (8)	0.0500 (7)	0.0481 (7)	−0.0154 (6)	−0.0102 (6)	−0.0058 (5)
C22	0.0899 (11)	0.0516 (8)	0.0517 (8)	−0.0157 (7)	−0.0138 (7)	0.0003 (6)
C23	0.0821 (10)	0.0590 (8)	0.0488 (7)	0.0073 (7)	−0.0205 (7)	−0.0096 (6)
C24	0.0532 (8)	0.0710 (9)	0.0558 (8)	0.0020 (7)	−0.0143 (6)	−0.0191 (7)
C25	0.0484 (7)	0.0551 (7)	0.0515 (7)	−0.0087 (6)	−0.0056 (5)	−0.0118 (6)
C26	0.0432 (6)	0.0429 (6)	0.0500 (6)	−0.0119 (5)	−0.0035 (5)	−0.0120 (5)
C27	0.0684 (9)	0.0546 (8)	0.0551 (8)	−0.0127 (6)	−0.0027 (6)	−0.0192 (6)
C28	0.0874 (11)	0.0704 (10)	0.0808 (11)	−0.0198 (8)	0.0051 (9)	−0.0426 (9)
C29	0.0809 (11)	0.0464 (8)	0.1152 (15)	−0.0155 (7)	0.0041 (10)	−0.0313 (9)
C30	0.0781 (11)	0.0430 (7)	0.0974 (12)	−0.0147 (7)	−0.0125 (9)	−0.0047 (8)
C31	0.0664 (9)	0.0458 (7)	0.0626 (8)	−0.0147 (6)	−0.0107 (6)	−0.0073 (6)

### Geometric parameters (Å, °)

**Table:**

N1—C6	1.3736 (14)	C16—C17	1.382 (2)
N1—N2	1.3778 (12)	C16—H16A	0.9300
N1—C5	1.3784 (14)	C17—H17A	0.9300
N2—C3	1.3286 (14)	O18—B19	1.5077 (15)
C3—C4	1.4221 (16)	B19—C26	1.6016 (17)
C3—C12	1.4695 (16)	B19—C20	1.6113 (17)
C4—C5	1.3599 (16)	C20—C21	1.3910 (17)
C4—H4A	0.9300	C20—C25	1.4003 (17)
C5—O18	1.3273 (13)	C21—C22	1.3862 (19)
C6—N7	1.3474 (13)	C21—H21A	0.9300
C6—C11	1.3900 (15)	C22—C23	1.377 (2)
N7—C8	1.3528 (14)	C22—H22A	0.9300
N7—B19	1.6412 (15)	C23—C24	1.376 (2)
C8—C9	1.3647 (17)	C23—H23A	0.9300
C8—H8A	0.9300	C24—C25	1.3799 (19)
C9—C10	1.3860 (17)	C24—H24A	0.9300
C9—H9A	0.9300	C25—H25A	0.9300
C10—C11	1.3715 (16)	C26—C27	1.3915 (17)
C10—H10A	0.9300	C26—C31	1.3940 (18)
C11—H11A	0.9300	C27—C28	1.3897 (19)
C12—C17	1.3857 (18)	C27—H27A	0.9300
C12—C13	1.3890 (18)	C28—C29	1.374 (3)
C13—C14	1.3815 (19)	C28—H28A	0.9300
C13—H13A	0.9300	C29—C30	1.370 (2)
C14—C15	1.381 (2)	C29—H29A	0.9300
C14—H14A	0.9300	C30—C31	1.3838 (19)
C15—C16	1.366 (2)	C30—H30A	0.9300
C15—H15A	0.9300	C31—H31A	0.9300
			
C6—N1—N2	121.20 (9)	C16—C17—C12	120.47 (14)
C6—N1—C5	126.52 (9)	C16—C17—H17A	119.8
N2—N1—C5	112.22 (9)	C12—C17—H17A	119.8
C3—N2—N1	103.56 (9)	C5—O18—B19	115.85 (8)
N2—C3—C4	112.44 (10)	O18—B19—C26	109.26 (9)
N2—C3—C12	118.30 (10)	O18—B19—C20	110.01 (9)
C4—C3—C12	129.22 (11)	C26—B19—C20	116.51 (9)
C5—C4—C3	105.22 (10)	O18—B19—N7	105.44 (8)
C5—C4—H4A	127.4	C26—B19—N7	107.22 (9)
C3—C4—H4A	127.4	C20—B19—N7	107.79 (9)
O18—C5—C4	134.33 (10)	C21—C20—C25	116.08 (11)
O18—C5—N1	118.96 (9)	C21—C20—B19	125.87 (11)
C4—C5—N1	106.55 (9)	C25—C20—B19	118.05 (10)
N7—C6—N1	115.43 (9)	C22—C21—C20	122.18 (13)
N7—C6—C11	122.21 (10)	C22—C21—H21A	118.9
N1—C6—C11	122.36 (10)	C20—C21—H21A	118.9
C6—N7—C8	118.15 (10)	C23—C22—C21	120.03 (14)
C6—N7—B19	119.99 (9)	C23—C22—H22A	120.0
C8—N7—B19	121.51 (9)	C21—C22—H22A	120.0
N7—C8—C9	122.52 (11)	C24—C23—C22	119.41 (13)
N7—C8—H8A	118.7	C24—C23—H23A	120.3
C9—C8—H8A	118.7	C22—C23—H23A	120.3
C8—C9—C10	118.84 (11)	C23—C24—C25	120.19 (13)
C8—C9—H9A	120.6	C23—C24—H24A	119.9
C10—C9—H9A	120.6	C25—C24—H24A	119.9
C11—C10—C9	119.84 (11)	C24—C25—C20	122.10 (13)
C11—C10—H10A	120.1	C24—C25—H25A	118.9
C9—C10—H10A	120.1	C20—C25—H25A	118.9
C10—C11—C6	118.42 (11)	C27—C26—C31	116.55 (12)
C10—C11—H11A	120.8	C27—C26—B19	122.56 (11)
C6—C11—H11A	120.8	C31—C26—B19	120.71 (11)
C17—C12—C13	118.37 (12)	C28—C27—C26	121.72 (14)
C17—C12—C3	121.57 (11)	C28—C27—H27A	119.1
C13—C12—C3	119.98 (11)	C26—C27—H27A	119.1
C14—C13—C12	120.83 (14)	C29—C28—C27	119.89 (15)
C14—C13—H13A	119.6	C29—C28—H28A	120.1
C12—C13—H13A	119.6	C27—C28—H28A	120.1
C15—C14—C13	119.88 (15)	C30—C29—C28	119.93 (14)
C15—C14—H14A	120.1	C30—C29—H29A	120.0
C13—C14—H14A	120.1	C28—C29—H29A	120.0
C16—C15—C14	119.73 (14)	C29—C30—C31	119.93 (15)
C16—C15—H15A	120.1	C29—C30—H30A	120.0
C14—C15—H15A	120.1	C31—C30—H30A	120.0
C15—C16—C17	120.67 (14)	C30—C31—C26	121.98 (14)
C15—C16—H16A	119.7	C30—C31—H31A	119.0
C17—C16—H16A	119.7	C26—C31—H31A	119.0
			
C6—N1—N2—C3	−178.54 (9)	C4—C5—O18—B19	149.85 (13)
C5—N1—N2—C3	−1.17 (12)	N1—C5—O18—B19	−35.58 (14)
N1—N2—C3—C4	0.83 (12)	C5—O18—B19—C26	162.66 (9)
N1—N2—C3—C12	−176.99 (9)	C5—O18—B19—C20	−68.27 (12)
N2—C3—C4—C5	−0.21 (13)	C5—O18—B19—N7	47.71 (12)
C12—C3—C4—C5	177.31 (11)	C6—N7—B19—O18	−34.70 (13)
C3—C4—C5—O18	174.52 (12)	C8—N7—B19—O18	152.14 (10)
C3—C4—C5—N1	−0.52 (12)	C6—N7—B19—C26	−151.05 (9)
C6—N1—C5—O18	2.33 (16)	C8—N7—B19—C26	35.80 (13)
N2—N1—C5—O18	−174.86 (9)	C6—N7—B19—C20	82.77 (11)
C6—N1—C5—C4	178.28 (10)	C8—N7—B19—C20	−90.38 (12)
N2—N1—C5—C4	1.08 (12)	O18—B19—C20—C21	117.35 (12)
N2—N1—C6—N7	−171.46 (9)	C26—B19—C20—C21	−117.62 (13)
C5—N1—C6—N7	11.57 (16)	N7—B19—C20—C21	2.88 (15)
N2—N1—C6—C11	8.11 (16)	O18—B19—C20—C25	−61.86 (13)
C5—N1—C6—C11	−168.85 (11)	C26—B19—C20—C25	63.16 (14)
N1—C6—N7—C8	−179.10 (9)	N7—B19—C20—C25	−176.34 (9)
C11—C6—N7—C8	1.32 (16)	C25—C20—C21—C22	0.30 (18)
N1—C6—N7—B19	7.52 (14)	B19—C20—C21—C22	−178.92 (12)
C11—C6—N7—B19	−172.06 (10)	C20—C21—C22—C23	−0.3 (2)
C6—N7—C8—C9	−1.63 (17)	C21—C22—C23—C24	−0.1 (2)
B19—N7—C8—C9	171.64 (11)	C22—C23—C24—C25	0.5 (2)
N7—C8—C9—C10	0.66 (19)	C23—C24—C25—C20	−0.6 (2)
C8—C9—C10—C11	0.64 (19)	C21—C20—C25—C24	0.15 (18)
C9—C10—C11—C6	−0.92 (18)	B19—C20—C25—C24	179.44 (11)
N7—C6—C11—C10	−0.07 (17)	O18—B19—C26—C27	145.00 (12)
N1—C6—C11—C10	−179.62 (10)	C20—B19—C26—C27	19.60 (17)
N2—C3—C12—C17	161.16 (11)	N7—B19—C26—C27	−101.21 (13)
C4—C3—C12—C17	−16.24 (19)	O18—B19—C26—C31	−40.18 (15)
N2—C3—C12—C13	−15.47 (17)	C20—B19—C26—C31	−165.58 (11)
C4—C3—C12—C13	167.14 (12)	N7—B19—C26—C31	73.61 (13)
C17—C12—C13—C14	−2.0 (2)	C31—C26—C27—C28	−0.9 (2)
C3—C12—C13—C14	174.76 (13)	B19—C26—C27—C28	174.12 (13)
C12—C13—C14—C15	0.1 (2)	C26—C27—C28—C29	0.0 (2)
C13—C14—C15—C16	1.6 (2)	C27—C28—C29—C30	0.6 (3)
C14—C15—C16—C17	−1.3 (2)	C28—C29—C30—C31	−0.3 (3)
C15—C16—C17—C12	−0.6 (2)	C29—C30—C31—C26	−0.6 (2)
C13—C12—C17—C16	2.2 (2)	C27—C26—C31—C30	1.2 (2)
C3—C12—C17—C16	−174.44 (12)	B19—C26—C31—C30	−173.89 (13)
